
JOURNAL INFORMATION
==============================
NLM Title Abbreviation: Biospektrum (Heidelb)
Journal ID: Biospektrum (Heidelb)
NLM Journal ID: 9615685

Biospektrum

ISSN: 0947-0867
EISSN: 1868-6249

ARTICLE INFORMATION
==============================
PMCID: PMC7880636
PMID: 33612990
DOI: 10.1007/s12268-021-1516-6
Article ID: 1516
Article version: 1
Subjects: Wissenschaft Special

Coronavirus-Replikation: Mechanismus und Inhibition durch Remdesivir

Cramer Patrick pcramer@mpibpc.mpg.de
1
Kokic Goran 1
Dienemann Christian 1
Höbartner Claudia 2
Hillen Hauke S. hauke.hillen@mpibpc.mpg.de
1 3
1 grid.418140.8 0000 0001 2104 4211 Abteilung Molekularbiologie, Max-Planck-Institut für biophysikalische Chemie, Am Fassberg 11, D-37077 Göttingen, Deutschland
2 grid.8379.5 0000 0001 1958 8658 Institut für Organische Chemie, Universität Würzburg, WüRzburg, Deutschland
3 grid.411984.1 0000 0001 0482 5331 Institut für Zellbiochemie, Universitätsmedizin Göttingen, Humboldtallee 23, D-37073 Göttingen, Deutschland
Electronic publication date: 2021 Feb 12
Print publication date: 2021
Volume: 27
Issue: 1
First page: 49
Last page: 53
Copyright: © Die Autoren 2021
License: Dieser Artikel wird unter der Creative Commons Namensnennung 4.0 International Lizenz veröffentlicht, welche die Nutzung, Vervielfältigung, Bearbeitung, Verbreitung und Wiedergabe in jeglichem Medium und Format erlaubt, sofern Sie den/die ursprünglichen Autor(en) und die Quelle ordnungsgemäß nennen, einen Link zur Creative Commons Lizenz beifügen und angeben, ob Änderungen vorgenommen wurden. Die in diesem Artikel enthaltenen Bilder und sonstiges Drittmaterial unterliegen ebenfalls der genannten Creative Commons Lizenz, sofern sich aus der Abbildungslegende nichts anderes ergibt. Sofern das betreffende Material nicht unter der genannten Creative Commons Lizenz steht und die betreffende Handlung nicht nach gesetzlichen Vorschriften erlaubt ist, ist für die oben aufgeführten Weiterverwendungen des Materials die Einwilligung des jeweiligen Rechteinhabers einzuholen. Weitere Details zur Lizenz entnehmen Sie bitte der Lizenzinformation auf http://creativecommons.org/licenses/by/4.0/deed.de.
License URL: https://creativecommons.org/licenses/by/4.0/

issue-copyright-statement: © Springer-Verlag GmbH Deutschland, ein Teil von Springer Nature 2021

==============================
Coronaviruses use an RNA-dependent RNA polymerase to replicate and transcribe their RNA genome. The structure of the SARS-CoV-2 polymerase was determined by cryo-electron microscopy within a short time in spring 2020. The structure explains how the viral enzyme synthesizes RNA and how it replicates the exceptionally large genome in a processive manner. The most recent structure-function studies further reveal the mechanism of polymerase inhibition by remdesivir, an approved drug for the treatment of COVID-19.

■ Wenn ein Coronavirus in eine Wirtszelle eindringt, wird das virale RNA-Genom in drei verschiedenen Prozessen verwendet [1]. Zunächst wird das Genom von der zellulären Translationsmaschinerie genutzt, um ein Polypeptid zu erzeugen. Dieses Polypeptid wird dann in verschiedene Proteine gespalten, die die virale Replikationsmaschinerie bilden. Danach wird das Genom repliziert, wozu zunächst eine komplementäre Negativstrang-RNA synthetisiert wird, mit deren Hilfe dann ein weiteres Positivstrang-Genom gebildet wird. Schließlich wird das virale RNA-Genom auch zur Herstellung komplementärer, sub-genomischer RNAs verwendet. Diese kürzeren RNAs werden als Matrizen für die Transkription genutzt, wodurch wiederum virale RNAs erzeugt werden, die der Proteinsynthese dienen. Diese drei Prozesse liegen der Produktion neuer Viren und somit der Eskalation der Infektion zugrunde.

Die Replikation des Coronavirus-Genoms wird von 16 viralen Proteinen bewerkstelligt, die als „nicht-strukturelle Proteine" (nsp1-nsp16) bezeichnet werden [2]. Diese Proteine umfassen u. a. die Protease nsp5, die zur Spaltung des anfänglichen langen Polypeptids benötigt wird und ein wichtiges Angriffsziel für antivirale Wirkstoffe darstellt. Die Proteine nsp7, nsp8 und nsp12 bilden die RNA-abhängige RNA-Polymerase (*RNA-dependent RNA polymerase*, RdRp). Diese Polymerase bewerkstelligt die Replikation und Transkription und ist das Wirkstoffziel des antiviralen Medikaments Remdesivir. Andere nicht-strukturelle Proteine sind für die RNA-Modifikation und die Fehlerkorrektur während der RNA-Synthese verantwortlich.

## Struktur der Coronavirus-Polymerase

Zu Beginn des Jahres 2020 wurde schnell klar, wie wichtig es ist, die Struktur und Funktion der Polymerase des neuen Coronavirus aufzuklären. Dies lag nicht nur deshalb auf der Hand, weil diese Polymerase von so zentraler Bedeutung für den Lebenszyklus des Coronavirus ist. Man wusste auch, dass Polymerasen anderer Viren bereits erfolgreich als Zielmoleküle für antivirale Medikamente genutzt werden. So wird die Polymerase des Herpes-Virus durch das Medikament Acyclovir gehemmt, und auch für die Polymerasen des AIDS-verursachenden HI-Virus und des Hepatitis-C-Virus gibt es therapeutisch eingesetzte Inhibitoren. All diese Wirkstoffe haben Ähnlichkeiten zu natürlichen RNA-Bausteinen, sodass sie von den

Hier steht eine Anzeige.

Springer

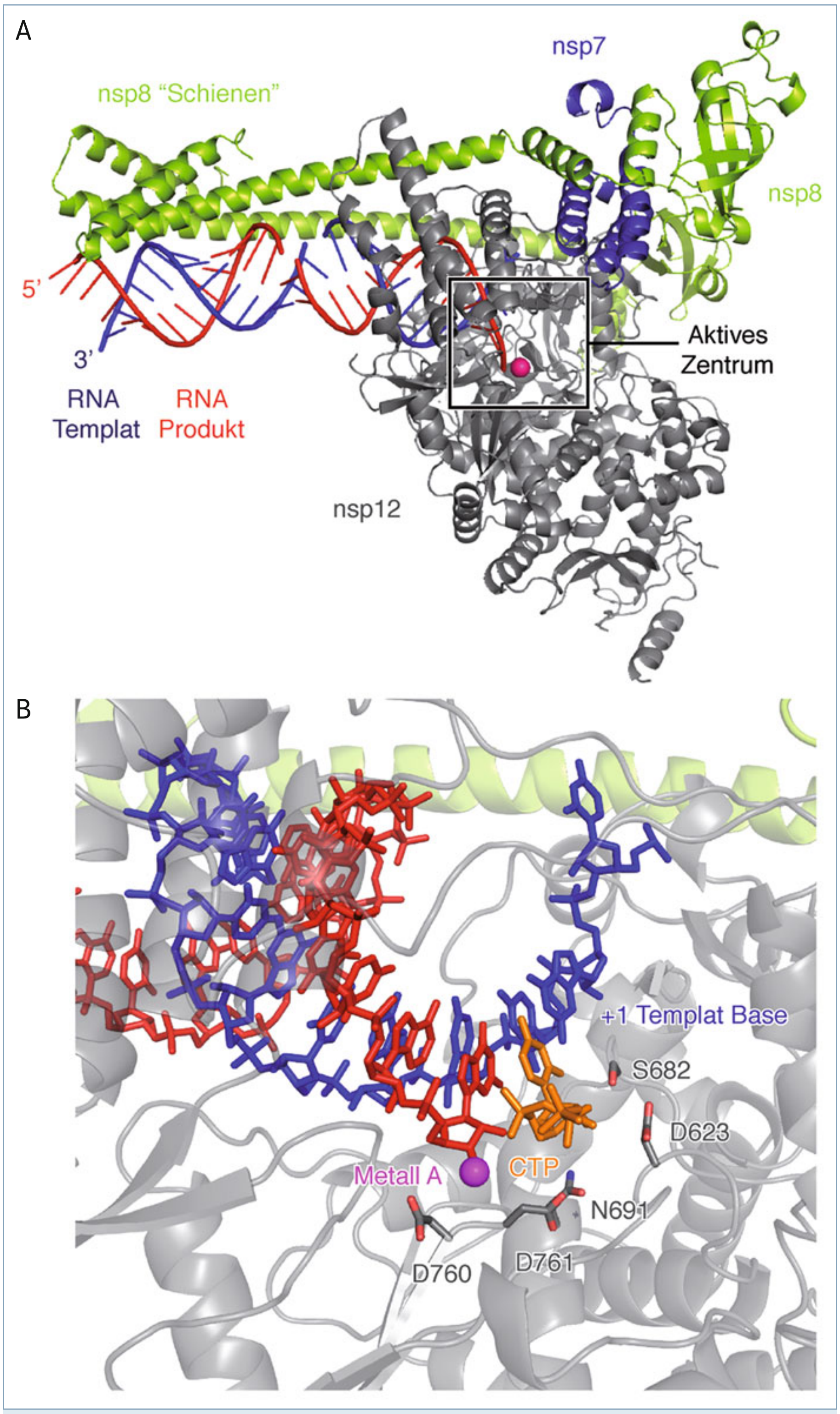

▲ **Abb. 1:** Struktur und Mechanismus der Coronavirus-Polymerase. **A**, Kryo-elektronenmikroskopische Struktur der RNA-abhängigen RNA-Polymerase des Coronavirus SARS-CoV-2 [6]. **B**, Mechanismus der RNA-abhängigen RNA-Synthese im aktiven Zentrum der Polymerase. Der RNA-Templat-Strang ist blau, das RNA-Produkt rot und das eintretende Nukleosid-Triphosphat (hier ein modelliertes CTP) orange dargestellt. Das Metallion A im aktiven Zentrum ist als magenta-farbene Kugel dargestellt. Für die Katalyse wichtige Seitenketten sind in Stabform dargestellt.

viralen Polymerasen in die wachsende RNA-Kette eingebaut werden, was in der Folge zu einer Störung der RNA-Synthese führt. Da unser Labor über Erfahrung in der biochemischen und strukturellen Analyse von Polymerasen verfügt [3–5], lag es nahe, umgehend die Struktur der Polymerase von SARS-CoV-2 aufzuklären.

So beschlossen wir im März 2020, dieses wichtige Projekt so schnell wie möglich umzusetzen. Bald kehrten fünf erfahrene Postdoktoranden aus dem Home-Office unter Hygieneauflagen ins Labor zurück. In wenigen Wochen konnte unsere „Task Force" die Struktur der SARS-CoV-2 RdRp im Komplex mit RNA durch Kryo-Elektronenmikroskopie lösen (**Abb. 1**, [6]). Zwar gab es einige Hürden zu überwinden. Doch die komplementäre Expertise der Teammitglieder brachte schnell den Erfolg. Wir konnten die RdRp nach heterologer Expression der Untereinheiten rekonstituieren und ihre Aktivität nachweisen. Eine Schwierigkeit ergab sich dann aber daraus, dass die Polymerase-Partikel im Mikroskop eine ähnliche Orientierung einnahmen, was eine vollständige Rekonstruktion in 3D zunächst verhinderte. Das bekamen wir allerdings durch Kippen der Probe und mit aufwendiger Datenprozessierung in den Griff. So konnten wir die Struktur bei einer nominalen Auflösung von 2,9 Å bestimmen. Das aktive Zentrum des Enzyms ließ sich bei einer noch höheren lokalen Auflösung von unter 2,5 Å abbilden, was chemische Details offenbarte.

## Mechanismus der viralen Replikation und *serendipity*

Die detaillierte Struktur der RdRp in aktiver Form umfasst die viralen Proteine nsp12, nsp8 und nsp7 und über zwei Windungen einer RNA-Doppelhelix, die aus Templat- und Produktstrang besteht (**Abb. 1**, [6]). Die Untereinheit nsp12 bildet eine Spalte, die die erste Windung der RNA-Duplex bindet und aus der die neu synthetisierte RNA austritt. Am Boden der Spalte befindet sich das aktive Zentrum der Polymerase, das aus konservierten Aminosäureresten besteht, die die RNA-Synthese katalysieren. All dies war nicht sehr verwunderlich im Vergleich zu bereits bekannten Coronavirus-Polymerasen. Spannend wurde die Strukturanalyse jedoch durch eine völlig unerwartete Beobachtung.

Im Gegensatz zu nsp12 und nsp7 liegt die Untereinheit nsp8 in zwei Kopien vor, die sich am Ausgang der nsp12-Spalte gegenüberliegen. Dort bilden die nsp8-Untereinheiten lange helikale Proteinbereiche aus, die wie Schienen entlang der RNA führen. An den Innenseiten dieser Schienen befinden sich positiv geladene Proteinbereiche, an denen die negativ geladene RNA während der Synthese entlanggleitet. Aus publizierten

Arbeiten [7] schlossen wir, dass diese langen nsp8-Schienen für die hohe Prozessivität der Polymerase verantwortlich sind. Als Prozessivität bezeichnet man die Eigenschaft der Polymerase, mit der RNA-Matrize assoziiert zu bleiben bis die Synthese erfolgreich abgeschlossen ist. Die hohe Prozessivität der Polymerase ist vermutlich für die Replikation des Coronavirus-Genoms unerlässlich, das mit rund 30.000 Nukleotiden das längste bekannte RNA-Genom darstellt [8].

Zeitgleich mit unserer Publikation veröffentlichten auch Forscherteams aus China RdRp-Strukturen des neuen Coronavirus [9–11]. Eine dieser Strukturen zeigte das freie Kern-Enzym [9], welches dem Kernteil unserer Struktur entspricht. Zwei weitere Strukturen [10, 11] enthielten RNA und ähnelten ebenfalls unseren Ergebnissen. In den Strukturen fehlten jedoch zumindest teilweise die von uns visualisierten nsp8-Schienen entlang der austretenden RNA. Der Grund, warum wir diese kritischen Elemente der Polymerase zum ersten Mal sahen, ist vermutlich, dass unsere Struktur eine lange RNA-Duplex enthielt, während in den anderen Strukturen entweder die RNA fehlte oder nur relativ kurze RNAs vorhanden waren. Auch wir hatten zunächst eine zu kurze RNA eingesetzt. Doch in der Eile hatten wir eine Sequenz verwendet, die sich spontan zu längeren RNA-Duplexen zusammenlagern konnte, die dann in der Struktur sichtbar wurden. Dieser glückliche Zufall – *serendipity* – unterstreicht, wie sinnvoll es ist, dass sich mehrere unabhängige Arbeitsgruppen mit der Untersuchung der Coronavirus-Mechanismen und der Entwicklung von Wirkstoffen befassen.

## Inhibition durch Remdesivir

Diese ersten Ergebnisse ermöglichten es, die Mechanismen von Virostatika zu untersuchen. Zuerst wollten wir den Mechanismus der Wirkung von Remdesivir aufklären. Remdesivir ist das erste Medikament, das in den USA und in der EU zur Notfallbehandlung von COVID-19-Patienten zugelassen wurde [12–14]. Die aktive Form von Remdesivir wirkt als Nukleosid-Analogon und hemmt die RdRp von Coronaviren einschließlich des neuen Virus SARS-CoV-2 [15]. Remdesivir ist ein Vorläufermolekül (*prodrug*), das in der Zelle zur aktiven Triphosphat-Spezies verstoffwechselt wird. Diese Verbindung wird dann anstelle von ATP für die RNA-Synthese durch die RdRp verwendet. So wird Remdesivir-Monophosphat in das wachsende RNA-Produkt eingebaut. Dies führt allerdings nicht zum Kettenabbruch, wie das bei vielen gängigen Nukleosid-Analoga der Fall ist. Vielmehr werden noch drei weitere Nukleotid-Bausteine in die wachsende RNA-Kette inkorporiert, bevor die RNA-Synthese zum Stillstand kommt [15].

Um den molekularen Mechanismus der Remdesivir-induzierten Inhibierung der Coronavirus-Polymerase aufzuklären, mussten wir synthetische RNA-Chemie mit Biochemie und Kryo-Elektronenmikroskopie kombinieren (**Abb. 2**, [16]). Wir bestätigten zunächst, dass die wachsende RNA-Kette Remdesivir und dann drei weitere Nukleotide einbauen kann. Allerdings entsteht dann eine Barriere für die weitere RNA-Translokation, die die Inkorporation eines vierten Nukleotids beeinträchtigt. Diese Barriere entsteht dadurch, dass eine zusätzliche chemische Gruppe, die sich an der Ribose der Remdesivir-Einheit befindet, bei der Translokation mit einer Serin-Seitenkette von nsp12 zusammen stößt. Die Translokations-Barriere bewirkt so den Verbleib des 3'-Nukleotids der RNA in der Substratbindungsstelle der Polymerase. Das wiederum stört die Bindung des nächsten Nukleosid-Triphosphats, das für die weitere RNA-Elongation benötigt wird, was die Inhibition der Polymerase erklärt. Unter bestimmten Bedingungen kann diese Barriere jedoch überwunden werden, sodass Remdesivir kein starker Inhibitor der RNA-Synthese ist.

## Zukünftige Arbeiten

Diese mechanistischen Erkenntnisse erleichtern die Suche nach verbesserten virostatischen Polymerase-Inhibitoren, die auf eine Unterdrückung der Coronavirus-Replikation abzielen. Von besonderer Bedeutung für die Wirkung von Polymerase-Inhibitoren ist das virale Protein nsp14. Nsp14 ist eine Exonuklease, die die Fehlerkorrektur während der RNA-Synthese ermöglicht [17]. Die Exonuklease schneidet fehlerhafte RNA-Produkte vom 3'-Ende her zurück, um so dem Effekt der Polymerase-Inhibitoren entgegenzuwirken [17]. Das 3'-Ende des RNA-Produktstrangs ist in der Struktur der Remdesivir-inhibierten Polymerase mit dem komplementären Templat-Nukleotid gepaart und vermutlich für die virale Exonuklease schwer zugänglich.

Hier steht eine Anzeige.
Springer

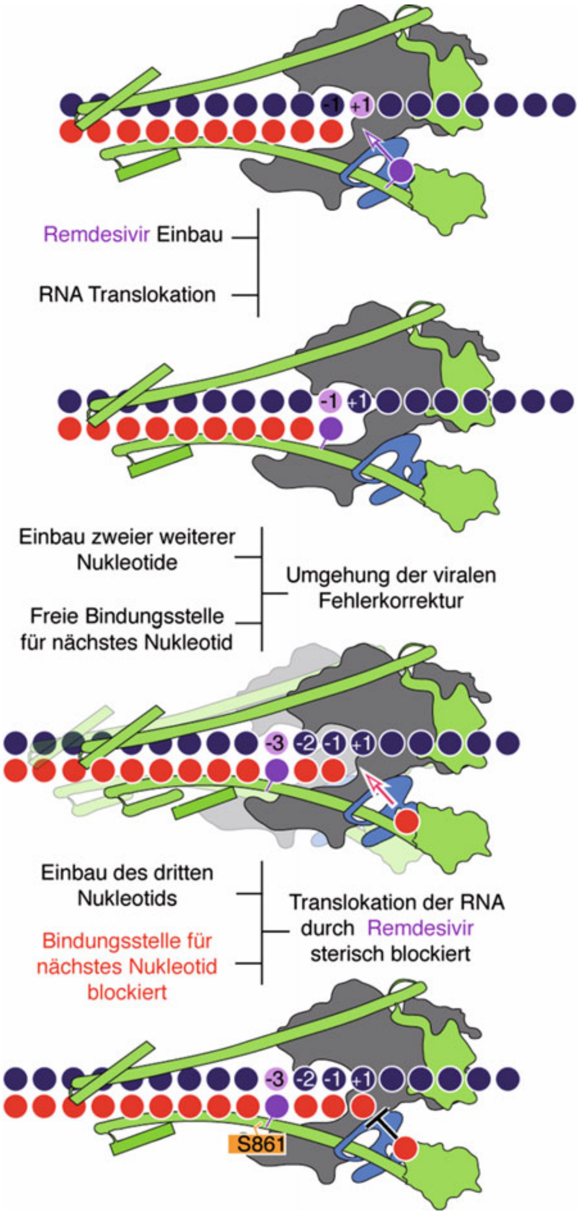

▲ **Abb. 2:** Inhibition der Coronavirus-Polymerase durch Remdesivir. Schematische Darstellung der Inkorporation von Remdesivir-Monophosphat und der Translokations-Barriere nach Einbau von drei weiteren Nukleotiden.

Somit scheint der Komplex vor einer effizienten Fehlerkorrektur geschützt, was wohl eine Voraussetzung für die Wirkung von Remdesivir darstellt. Allerdings findet die Fehlerkorrektur trotzdem in geringem Umfang statt und macht Remdesivir weniger effizient. In Zukunft sollten daher die molekularen Mechanismen der viralen Fehlerkorrektur untersucht werden, bei denen auch die virale Helikase nsp13 eine Rolle spielen könnte [18].

Parallel dazu werden wir und andere Gruppen nach neuartigen Polymerase-Inhibitoren suchen. Dabei hoffen wir Leitsubstanzen zu finden, die einen anderen Wirkmechanismus aufweisen als Remdesivir. Das Ziel wird es sein, Verbindungen zu entwickeln, die selektiv nur die virale Polymerase inhibieren, aber auf die zellulären RNA-Polymerasen keinen Einfluss haben. Solche Leitsubstanzen sind dann gute Kandidaten für eine weitere Entwicklung als Virostatika in Zellkultur und im Modellorganismus. Dieser Ansatz birgt die Hoffnung in sich, eines Tages gut wirksame Medikamente gegen Coronavirus-Infektionen in den Händen zu halten. Virostatische Medikamente werden zusätzlich zu sicheren und wirksamen Impfstoffen nach wie vor benötigt. Durch solche Virostatika ließen sich lokale Ausbrüche schnell kontrollieren, das Umfeld von Infizierten prophylaktisch schützen und zukünftige Infektionen mit neuartigen Coronaviren eindämmen. ■

## AUTOREN

Hier steht eine Anzeige.
Springer


Funding note

Open Access funding enabled and organized by Projekt DEAL.

Patrick Cramer 1989–1995 Chemiestudium an den Universitäten Stuttgart, Heidelberg, Bristol, Cambridge. 1995–1998 Promotion am EMBL Grenoble, Frankreich. 1999–2001 Postdoktorand an der Stanford University, CA, USA. 2001–2014 Professor an der LMU München. Seit 2014 Direktor am Max-Planck-Institut für Biophysikalische Chemie in Göttingen.

Goran Kokic 2007–2012 Studium der Molekularen Biologie an der Universität Zagreb, Kroatien. 2013–2019 Masterstudium und Promotion in der International Max-Planck Research School for Molecular Biology und am Max-Planck-Institut für Biophysikalische Chemie, Göttingen. Seit 2019 Postdoktorand am Max-Planck-Institut für Biophysikalische Chemie.

Christian Dienemann 2008–2013 Studium der Molekularbiologie und Biochemie an den Universitäten Jena und Aarhus, Dänemark. 2013–2018 Promotion an der Universität Göttingen. Seit 2018 Wissenschaftler für Kryo-Elektronenmikroskopie am Max-Planck-Institut für Biophysikalische Chemie in Göttingen.

Claudia Höbartner 1995–2001 Chemiestudium an den Universitäten Wien, Österreich, und Zürich, Schweiz. 2001–2004 Promotion an der Universität Innsbruck. 2005–2007 Postdoktorat an der University of Illinois, USA. 2008–2014 Gruppenleiterin am Max-Planck-Institut für Biophysikalische Chemie in Göttingen. 2014–2017 Professorin an der Universität Göttingen. Seit 2017 Professorin an der Universität Würzburg.

Hauke S. Hillen 2007–2013 Biochemiestudium an den Universitäten Tübingen und Berkeley, CA, USA. 2013–2017 Promotion an der LMU München. 2018–2020 Projektleiter am Max-Planck-Institut für Biophysikalische Chemie, Göttingen. Seit 2020 Juniorprofessor an der Universitätsmedizin Göttingen sowie Forschungsgruppenleiter am Max-Planck-Institut für Biophysikalische Chemie, Göttingen.


Literatur

[1] Hilgenfeld R Peiris M From SARS to MERS: 10 years of research on highly pathogenic human coronaviruses Antiviral Res 2013 100 286 295 10.1016/j.antiviral.2013.08.015 24012996 PMC7113673
[2] Snijder EJ Decroly E Ziebuhr J The nonstructural proteins directing coronavirus RNA synthesis and processing Adv Virus Res 2016 96 59 126 10.1016/bs.aivir.2016.08.008 27712628 PMC7112286
[3] Osman S Cramer P Structural biology of RNA polymerase II transcription: 20 years On Annu Rev Cell Dev Biol 2020 36 1 34 10.1146/annurev-cellbio-042020-021954 32822539
[4] Cramer P Organization and regulation of gene transcription Nature 2019 573 45 54 10.1038/s41586-019-1517-4 31462772
[5] Cramer P Eukaryotic transcription turns 50 Cell 2019 179 808 812 10.1016/j.cell.2019.09.018 31675494
[6] Hillen HS Kokic G Farnung L Structure of replicating SARS-CoV-2 polymerase Nature 2020 584 154 156 10.1038/s41586-020-2368-8 32438371
[7] Subissi L Posthuma CC Collet A One severe acute respiratory syndrome coronavirus protein complex integrates processive RNA polymerase and exonuclease activities Proc Natl Acad Sci U S A 2014 111 E3900 3909 10.1073/pnas.1323705111 25197083 PMC4169972
[8] Posthuma CC Te V A Snijder EJ Nidovirus RNA polymerases: complex enzymes handling exceptional RNA genomes Virus Res 2017 234 58 73 10.1016/j.virusres.2017.01.023 28174054 PMC7114556
[9] Gao Y Yan L Huang Y Structure of the RNA-dependent RNA polymerase from COVID-19 virus Science 2020 368 779 782 10.1126/science.abb7498 32277040 PMC7164392
[10] Yin W Mao C Luan X Structural basis for inhibition of the RNA-dependent RNA polymerase from SARS-CoV-2 by remdesivir Science 2020 368 1499 1504 10.1126/science.abc1560 32358203 PMC7199908
[11] Wang Q Wu J Wang H Structural basis for RNA replication by the SARS-CoV-2 polymerase Cell 2020 182 417 428 10.1016/j.cell.2020.05.034 32526208 PMC7242921
[12] Beigel JH Thomashek KM Dodd LE Remdesivir for the treatment of Covid-19—preliminary report N Engl J Med 2020 383 1813 1826 10.1056/NEJMoa2007764 32649078
[13] Grein J Ohmagari N Shin D Compassionate use of remdesivir for patients with severe Covid-19 N Engl J Med 2020 382 2327 2336 10.1056/NEJMoa2007016 32275812 PMC7169476
[14] Pan H, Peto R, Henao-Restrepo AM et al. (2020) Repurposed antiviral drugs for COVID-19; interim WHO SOLIDARITY trial results. N Engl J Med, doi: 10.1056/NEJMoa202318410.1056/NEJMoa2023184 PMC7727327 33264556
[15] Gordon CJ Tchesnokov EP Woolner E Remdesivir is a direct-acting antiviral that inhibits RNA-dependent RNA polymerase from severe acute respiratory syndrome coronavirus 2 with high potency J Biol Chem 2020 295 6785 6797 10.1074/jbc.RA120.013679 32284326 PMC7242698
[16] Kokic G Hillen HS Tegunov D Mechanism of SARS-CoV-2 polymerase inhibition by remdesivir Nat Commun 2020 12 279 10.1038/s41467-020-20542-0 PMC7804290 33436624
[17] Robson F Khan KS Le TK Coronavirus RNA proofreading: molecular basis and therapeutic targeting Mol Cell 2020 79 710 727 10.1016/j.molcel.2020.07.027 32853546 PMC7402271
[18] Chen J Malone B Llewellyn E Structural basis for helicase-polymerase coupling in the SARS-CoV-2 replication-transcription complex Cell 2020 182 1560 1573 10.1016/j.cell.2020.07.033 32783916 PMC7386476
